# CO Oxidation at Near-Ambient Temperatures over TiO_2_-Supported Pd-Cu Catalysts: Promoting Effect of Pd-Cu Nanointerface and TiO_2_ Morphology

**DOI:** 10.3390/nano11071675

**Published:** 2021-06-25

**Authors:** Abdallah F. Zedan, Safa Gaber, Amina S. AlJaber, Kyriaki Polychronopoulou

**Affiliations:** 1 National Institute of Laser Enhanced Science, Cairo University, Main Campus, Giza 12613, Egypt; 2 Center for Catalysis and Separations, Khalifa University of Science and Technology, Abu Dhabi P.O. Box 127788, United Arab Emirates; saagaber90@gmail.com; 3 Department of Chemistry, Qatar University, Doha 2713, Qatar; a.s.aljaber@qu.edu.qa; 4 Department of Mechanical Engineering, Khalifa University of Science and Technology, Abu Dhabi P.O. Box 127788, United Arab Emirates

**Keywords:** heterogeneous catalysis, carbon monoxide, palladium, copper, titania, nanointerface, morphology

## Abstract

Significant improvement of the catalytic activity of palladium-based catalysts toward carbon monoxide (CO) oxidation reaction has been achieved through alloying and using different support materials. This work demonstrates the promoting effects of the nanointerface and the morphological features of the support on the CO oxidation reaction using a Pd-Cu/TiO_2_ catalyst. Pd-Cu catalysts supported on TiO_2_ were synthesized with wet chemical approaches and their catalytic activities for CO oxidation reaction were evaluated. The physicochemical properties of the prepared catalysts were studied using standard characterization tools including SEM, EDX, XRD, XPS, and Raman. The effects of the nanointerface between Pd and Cu and the morphology of the TiO_2_ support were investigated using three different-shaped TiO_2_ nanoparticles, namely spheres, nanotubes, and nanowires. The Pd catalysts that are modified through nanointerfacing with Cu and supported on TiO_2_ nanowires demonstrated the highest CO oxidation rates, reaching 100% CO conversion at temperature regime down to near-ambient temperatures of ~45 °C, compared to 70 °C and 150 °C in the case of pure Pd and pure Cu counterpart catalysts on the same support, respectively. The optimized Pd-Cu/TiO_2_ nanowires nanostructured system could serve as efficient and durable catalyst for CO oxidation at near-ambient temperature.

## 1. Introduction

The catalytic oxidation of carbon monoxide (CO) to carbon dioxide (CO_2_) over supported metal catalysts has been considered one of the most efficient technologies to remove toxic CO pollutants from the environment [1,2,3,4]. Among the different studied catalytic systems, palladium-based nano catalysts have shown excellent catalytic activity for the CO oxidation reaction in the absence and presence of hydrogen [5,6,7,8]. However, palladium by itself is an expensive and rare metal. Therefore, great research efforts have been devoted to the improvement of the catalytic activity and stability of the Pd-based catalysts toward the low-temperature CO oxidation [9]. To achieve this goal, several strategies have been investigated including alloying [10,11] or coupling different plasmonic materials, development of bimetallic catalysts [12] using different support materials [9,12] hence increasing the porosity of the support [13,14] and improving the metal–support interaction [10,15] as well as nanointerfacing. The metal–support interaction was found to contribute to higher catalytic activity through enhanced metal dispersion and stability. At the same time, establishing nanointerface between Pd and other metals has been shown as a promising way for improving the catalytic activity toward CO oxidation at low or ambient temperatures [16,17]. When it comes to the support, TiO_2_-based nanomaterials have been widely used as catalytic support in several environmental applications including CO removal [1,18,19]. The TiO_2_ support is known for its high surface area, outstanding chemical stability under reducing and oxidizing conditions, and capability to provide an oxygen-transport medium in the gas-solid reactions [20,21,22].

This study focuses on the rational synthesis of TiO_2_-supported Pd-Cu catalysts and modulating their associated CO conversion temperatures by tailoring the Pd-Cu interface and the TiO_2_ morphology for enhanced activity in CO oxidation. The synthesis of the active Pd-based catalysts was based on a two-step wet impregnation of the Pd and Cu nanoparticles onto previously prepared TiO_2_ support of three different morphologies, namely nearly nanospheres, nanotubes, and nanowires to probe the effects of the Pd-Cu interface and the TiO_2_ properties on the surface reactivity and CO oxidation rates. Here, we demonstrate that Pd-Cu nano interfacing while using TiO_2_ support could increase the metal–support interaction and enhance the catalytic activity for CO oxidation at near-ambient temperatures and thus raising the catalyst’s potential in practical applications. 

## 2. Materials and Methods 

### 2.1. Materials

Chemicals and reagents were purchased from Sigma-Aldrich (Taufkirchen, Germany) and used without further purification, including titanium (IV) oxide spherical particles (TNS, anatase, −325 mesh powder, ≥99% trace metals basis, Sigma-Aldrich), sodium hydroxide (pellets, anhydrous, ≥98%, Sigma-Aldrich), potassium hydroxide (reagent grade, ≥98%, Sigma-Aldrich), hydrochloric acid (ACS reagent, 37%, Sigma-Aldrich), copper (II) nitrate trihydrate (purum, 98%, Sigma-Aldrich), ammonium carbonate (ACS reagent, 99%, Sigma-Aldrich), and palladium (II) nitrate solution 10 wt. % in 10 wt. % nitric acid (99.99% trace metal basis, Sigma-Aldrich). The water used was ultrapure deionized water (Direct-Q 5UV/Millipore, Molsheim, France). 

### 2.2. Methods

#### 2.2.1. Synthesis of Titania Nanotubes (TNT) and Titania Nanowires (TNW) 

The titania (TiO_2_) nanotubes (TNT) and nanowires (TNW) were prepared by the strong alkaline treatment of spherical anatase TiO_2_ particles under hydrothermal conditions followed by the acidic treatment using diluted HCl solution, as reported earlier [19,23]. 

Titania nanotubes (TNT): For the synthesis of titania nanotubes (TNT), 2 g of TiO_2_ anatase spherical (TNS) particles were mixed with 40 mL of 10 M aqueous NaOH solution in a 100 mL Teflon vessel. The mixture was stirred for 30 min at ambient temperature and pressure. The Teflon vessel containing the mixture was placed inside a stainless-steel autoclave reactor and was then transferred to an electric oven (Isotemp, Fisher-Scientific, Marietta, OH, USA). The autoclave reactor was kept in the electric oven at 140 °C for 48 h. Upon completion, the hydrothermal products were separated by filtration and rinsed thoroughly with deionized water several times. Then, the products were soaked in an aqueous solution for 2 h at room temperature after adjusting the pH to 6 with 0.1 M HCl. Finally, the products were separated by filtration and were dried in an electric oven at 80 °C for 24 h. The dried powder samples were calcined in a muffle furnace (Nebertherm, Bremen, Germany) under static air at 450 °C for 2 h with a heating rate of 5° min^−1^. 

Titania nanowires (TNW): For the synthesis of the titania nanowires (TNW), a procedure typical to that used for the synthesis of TNT was followed except using 10 M aqueous potassium hydroxide (KOH) as an alkaline agent for the hydrothermal treatment. All other steps including separation, purification, acidic treatment, drying, and calcination were the same as the TNT samples. The TiO_2_ different shaped supports are coded as TNS, TNT, and TNW in the following sections.

#### 2.2.2. Synthesis of Copper/Titania (Cu/TiO_2_) Catalysts

The copper/titania catalysts with different-shaped titania supports were generally prepared by the homogenous deposition precipitation using ammonium carbonate as a precipitating agent and followed by high-temperature calcination of purified and dried products. The different Cu/TiO_2_ catalysts were synthesized using the same loading of 2 wt. % Cu on TiO_2_ of various shapes (nanospheres, nanotubes, and nanowires). The catalysts that were prepared were namely 2 wt. % Cu/TNS, 2 wt. % Cu/TNT, and 2 wt. % Cu/TNW. In a typical synthesis of each of the Cu/TNS, Cu/TNT, and Cu/TNW, about 600 mg of TNS, TNT, and TNW were separately dispersed in a 40 mL deionized water by stirring and ultrasonication in an Erlenmeyer flask. A pre-determined volume of an aqueous solution of copper II nitrate-trihydrate (Cu (NO_3_)_2_.3H_2_O) was added to the respective titania-water suspension to give a final nominal loading of 2 wt. % Cu on various-shaped titania supports. The aqueous Cu^2+^/TiO_2_ reaction mixture was aged at ambient temperature for 12 h under continuous mechanical stirring followed by a drop-wise addition of an aqueous solution of 0.5 M ammonium carbonate ((NH_4_)_2_CO_3_) till the pH of the mixture reached ~9. Then the reaction mixture was stirred under mild temperature of 70 °C for 6 h. The resulting precipitated products were separated by centrifugation and were washed three times with deionized water. Finally, the obtained precipitates were dried at 80 °C for 12 h followed by calcination in a closed muffle furnace at 400 °C for 2 h with a heating ramp rate of 5° min^−1^. 

#### 2.2.3. Synthesis of Palladium-Copper/Titania (Pd-Cu/TiO_2_) Catalysts 

The different palladium-copper/titania catalysts were prepared using the controlled deposition precipitation method followed by the high-temperature calcination. The different palladium-copper/titania catalysts contained fixed amounts of 5 wt. % Pd and 2 wt. % Cu loaded onto various-shaped TiO_2_ (nanospheres, nanotubes, and nanowires) particles. The prepared palladium-copper/titania catalysts were coded as Pd-Cu/TNS, Pd-Cu/TNT, and Pd-Cu/TNW. These catalysts were generally synthesized as follows: for each catalyst, about 0.5 g of Cu/TNS, Cu/TNT, and Cu/TNW was separately suspended in a 50 mL deionized water by stirring and ultrasonication. Then, a pre-determined volume of palladium (II) nitrate solution 10 wt. % in 10 wt. % nitric acid (Pd (NO_3_)_2_/HNO_3_) was added to the respective Cu/titania aqueous suspension to give a final nominal loading of 5 wt. % Pd on various-shaped 2% Cu/titania. The aqueous Pd^2+^/Cu/TiO_2_ reaction mixture was aged at ambient temperature for 12 h under continuous mechanical stirring after a drop-wise addition of an aqueous solution of 0.2 M sodium hydroxide ((NaOH) till the pH of the mixture reached ~9. The resulting precipitated products were separated by centrifugation and were washed three times with deionized water. The obtained precipitates were dried at 80 °C for 12 h and were finally calcined in a closed muffle furnace at 400 °C for 2 h with a heating ramp rate of 5° min^−1^ to obtain the powder catalysts. For the sake of comparison different Pd/titania catalysts with fixed loading of 5 wt. % Pd on various-shaped TiO_2_ particles (Pd/TNS, Pd/TNT, and Pd/TNW) were prepared using the above-mentioned procedure. The deposition precipitation of Pd was performed over pure TiO_2_ nanospheres, nanotubes, and nanowires instead of copper/titania particles. The same procedure for purification and calcination was followed. All prepared calcined catalysts were kept for characterization and further catalysis measurements. 

### 2.3. Catalysts Characterization

The prepared catalysts were characterized using different techniques as it is described below. 

NOVA NANOSEM 450 (FEI, Brno, Czech Republic) was used to collect scanning electron microscopy (SEM) images. The sample under study was coated with thin layer of gold to avoid charging effect. X-ray diffraction (XRD) spectra were collected using MiniFlex II powder diffraction system (Rigaku, Tokyo, Japan) with Cu Kα radiation (λ = 1.54056 Å). The analysis was done with diffractometer at a voltage of 30 kV and intensity of 20 mA with scanning speed of 0.025°/step/second. The average crystallite size was calculated using Scherrer equation $D=\frac{k\;\lambda }{\beta \cos\theta },\;$ where D indicates the average crystallite size, K = 0.9 (shape factor), λ is the X-ray wavelength, θ is the X-ray reflection angle, and β is the full width at half-maximum (FWHM) of the diffraction peak (radians) [24,25,26]. Nitrogen adsorption and desorption isotherms were collected on ASAP 2460 pore size analyzer (Micromeritics, Norcross, GA, USA) (at 77 K) and recorded in the range of 0.05 to 1 relative pressure. The specific surface area was calculated based on the BET (Brunauer-Emmett-Teller) method. The samples were degassed at 90 °C for 1 h then at 150 °C for 1 h using N_2_-He mixture before the physisorption analysis. X-ray photoelectron spectroscopy (XPS) analysis was performed to give an insight to the oxidation state and chemical environment at the surface of the catalyst. KRATOS AXIS Ultra XPS (KRATOS Analytical, Manchester, UK) spectroscopy was used and equipped with a monochromatic Al Kα radiation source (hv = 1486.6 eV) in chamber at ca. 5 × 10^−9^ Torr base pressure. The high-resolution core level spectra measurement was done at high-resolution pass energy of 20 eV, 10 mA emission current, and 15 kV anode HT. The C1s peak was used for calibration and correcting the surface charging effect. Raman analysis was conducted using 780 nm laser sources in DXR 2 Raman Microscope (Thermo-Fisher Scientific, Madison, WI, USA). The analysis was done at 5 mW power and 4 cm^−1^ spectral resolution.

### 2.4. Catalytic Activity and Stability

The catalytic activity of the prepared catalysts was evaluated toward the CO oxidation reaction. A fixed bed catalytic reactor including a quartz tube of 10 mm inner diameter was used. The details of CO oxidation experiments are described in our previous works [4,27,28]. In each experiment, 50 mg catalyst was loaded and then placed in the middle of a programmable split tube furnace (Lindberg/Blue M Mini-Mite, Thermo Fisher Scientific, Waltham, MA, USA) under atmospheric pressure. In a typical catalysis measurement, a feed gas comprising 4% CO and 20% O_2_ (with Ar as a balance) and having a flow rate of 60 cm^3^/min (72,000 cm^3^ g^−1^ h^−1^ WHSV) was introduced into the bed. Effluents of the bed were analyzed using an infrared gas (IR) analyzer (IR200, Yokogawa, Japan). The surface temperature of the catalyst bed was raised with 5 °C/min ramping and monitored through in situ *k*-type thermocouple. The percentage volume of the CO and O_2_ as reactants, CO_2_ as a product as well as the temperature were recorded simultaneously using custom-configured program based on the LabVIEW data acquisition software (Version 14.0F1, National Instruments, Austin, TX, USA). The long-term stability of the selected catalyst was evaluated at 60 ± 5 °C for 72 h under continuous feed gas. The percentage of CO conversion was calculated from the equation, $\;X_{\mathrm{CO}}\;\left(\%\right)=\frac{{\left[\mathrm{CO}\right]}_{\mathrm{in}}-{\left[\mathrm{CO}\right]}_{\mathrm{out}}}{{\left[\mathrm{CO}\right]}_{\mathrm{in}}}\;\times \;100$, where *X* is the percentage conversion and [CO] is the CO molar flow in the inlet/ outlet feed gas.

## 3. Results and Discussion

### 3.1. Morphological Features 

**Figure 1:**
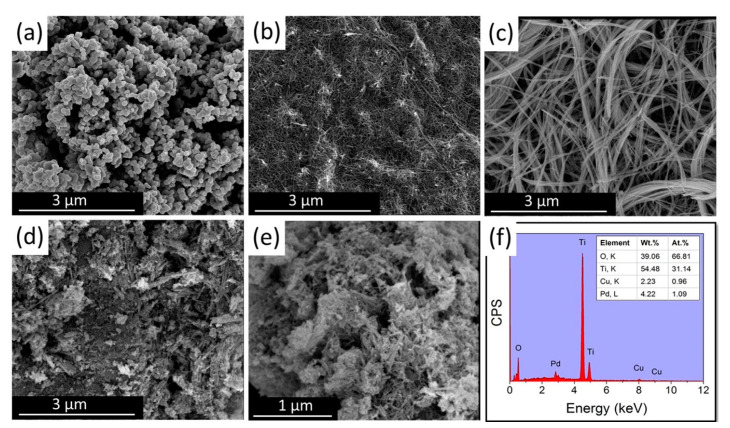
SEM images of (**a**) TiO_2_ sphere; (**b**) TiO_2_ nanotube; (**c**) TiO_2_ nanowire; (**d**,**e**) Pd-Cu–TiO_2_ nanowire catalyst; and (**f**) elemental analysis of Pd-Cu/TiO_2_ nanowire catalyst.

Scanning electron microscopy (NOVA NANOSEM 450, Brno, Czech Republic) was used to observe the morphology of the different TiO_2_ supports and the influence of the surface morphology on the Pd-Cu incorporation. Figure 1 shows the morphology of (a) TiO_2_ sphere (b) TiO_2_ nanotube, (c) TiO_2_ nanowire, and (d-e) Pd-Cu–TiO_2_, nanowire. The TiO_2_ sphere (Figure 1a) are loosely agglomerating with an average particle size of around 115 nm. Figure 1b shows a uniform nanotubular morphology with an average diameter of 29 nm and few micrometer lengths. The nanowire (Figure 1c) shows an ultra-fine well separated wire morphology with a diameter of ~45 nm and a length of several micrometers. Comparing (Figure 1c) with (Figure 1d,e), it can be seen that the nanowire morphology changed after Pd and Cu addition, where the wire length became shorter adopting a more flake-like morphology. It was possible to identify the elements present in the catalytic system through EDX analysis (Figure 1f), where peaks associated with Cu, Pd, Ti, and O in absence of any other impurity were found. In addition, from the measured elemental composition analysis, the loaded metals were identified to be 4.2 wt.% for Pd and 2.2 wt.% for Cu which is close enough to the theoretical (nominal) metal loading (5 wt.% for Pd and 2 wt.% for Cu). This shows that no loss, due to e.g., leaching, happened during the synthesis and/or the calcination steps. Regarding the slight excess of O_2_ compared to the wt.% (EDX) required by TiO_2_, this could be due to the presence of Pd and Cu oxide. In addition, it should be kept in mind that EDX is not quite ideal for the quantification of light elements like oxygen, so some contribution of the intrinsic deficiency of the EDX technique cannot be excluded. 

### 3.2. Crystal Structure

**Figure 2:**
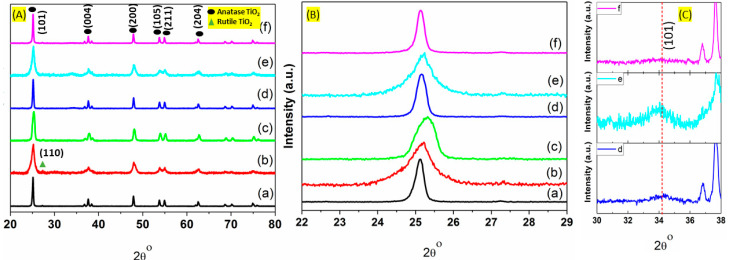
(**A**) XRD patterns of (a) Cu-TNS, (b) Cu-TNT, (c) Cu-TNW, (d) Pd-Cu-TNS, (e) Pd-Cu/TNT, (f) Pd-Cu/TNW catalysts. (**B**) TiO_2_ anatase (101) reflection of the catalysts in (**A**); (**C**) PdO (101) reflection for the (d–f) catalysts of (**A**).

The crystallinity of the different catalysts was investigated using X-ray diffraction and the results are presented in Figure 2A. As it can be seen, all the major characteristic peaks coincide well with TiO_2_ anatase phase. In particular, the main peaks at around 25.3, 37.6, 47.9, 53.7, 55.0, and 62.2 2theta values can be assigned to (101), (004), (200), (105), (211), and (204) reflections of the anatase phase, respectively. All these peaks correlate well with the Joint Committee on Powder Diffraction standard (JCPDS No. 21-1272) for the TiO_2_ anatase phase [29,30,31]. In general, in all of the prepared catalysts, the crystal structure was maintained after mono (Cu or Pd) and bimetallic (Cu-Pd) addition on the titanium oxide support, in any of the studied morphologies, was performed. Comparing the XRD pattern with literature and our previously reported XRD pattern of anatase TiO_2_, it can be found that addition of Cu on the different supports, does not result in any CuO peaks formation. The XRD patterns show no distinguished diffraction peak for CuO, metallic phase of Cu or Pd or their Pd-Cu alloy, maybe due to the fact that the studied metal loading is relatively low (5 wt. % Pd and 2 wt. % Cu) or the final size is too small to be detected. The presence of a small peak at 2θ = 27.32°, in some cases, such as in the Cu/TNS catalyst, has been linked to rutile phase impurities as it corresponds to (110) crystal plane of rutile phase of titanium oxide (JCPDS Card no. 21-1276) [29,32]. In all of the herein prepared catalysts, a slight upshift was found in the main (101) 2θ value peak compared to pure TiO_2_ (Figure 2B). Similar shift was reported by Ahmad et al. in their study of bimetallic palladium-supported halloysite nanotubes [12]. This shift can be attributed to the decrease in the values of lattice constant (lattice unit cell reduction) upon partial metal incorporation that cannot be excluded [12].

The crystallite sizes of anatase TiO_2_ were determined based on the diffraction peak at (101) plane using Scherrer’s equation (Figure 2B). The results are summarized in Table 1 and were found to be 29.5 nm, 9.2 nm, and 14.1 nm for Cu/TNS, Cu/TNT, and Cu/TNW, respectively. The further addition of the second metal, Pd, to the system results in an increase in the relative crystallite size in titanium nanotube and nanowire morphology cases, as the size increases to 13.9 nm for Cu/TNT and up to 32.1 nm for Cu/TNW. Yao et al. reported similar observation in their study of CuO addition to the outer surface of titanium oxide nanotube [33]. However, it is worth mentioning that the crystallite size increases largely in the titanium oxide with nanowire morphology (TNW case). As a result, the width of the peak assigned to the (101) plane becomes narrow and sharp when Pd was introduced. This can indicate that Pd addition can induce structural and surface changes; the latter can vary with the structural morphology of the support. Mostly, it is believed that Pd addition may display a preferential growth along the in-plane axis of the nanowire TiO_2_. On the other hand, the addition of Pd to the titanium oxide nanosphere (TNS case) resulted in slight decrease in the crystallite size (27.6 nm), may be due to the crystal growth interruption by the Pd introduction on the titanium oxide sphere. A decrease in crystallite size upon doping of different metals, Cu^2+^, Zn^2+^, and Fe^2+^ in the titanium oxide was previously reported [34,35]. In general, we can reasonably conclude that different morphologies induce different response in the system upon Pd addition, due to varying solution species diffusivity and differences in the solute species solid surface contact area, spheres, nanowires, and nanotubes are expected to have different contact areas for the anchoring of the species coming from the solution. This process is expected to lead to different metal-support interface/interactions, affecting the subsequent catalysis taking place.

Compared to the monometallic systems, the presence of palladium in the Cu/TiO_2_ catalyst (bimetallic system) leads to the appearance of a small peak at around 2θ = 34.4°as can be seen in (Figure 2C). This peak can be assigned to (101) reflection of PdO phase (PdO, JCPDS 41-1107) [36,37]. Using Scherrer’s equation, the crystallite size of PdO was found to be ~6.7 nm. The PdO peaks appearance can be attributed to the fact that Cu presence is not expected to facilitate the complete reduction of Pd^2+^ to Pd^0^, in the absence of a reducing agent (e.g., hydrogen) in the bimetallic TiO_2_ catalysts. This explanation agrees well with similar studies on bimetallic photocatalysts Pd–Cu/TiO_2_ by P. Lisowski and his group [38]. However, it should be noted that this is not the case for Pd/Cu on TNW where the PdO peak almost disappear (see Figure 2C). This could be explained by the fact that nanowire TiO_2_ has high surface to volume ratio and long axial morphology which mean more exposed surface available for Pd-Cu interaction which is expected to facilitate the reduction of Pd^2+^ [39]. This nano interfacial morphology leads to well dispersion of the Pd metal on the crystal lattice of titanium oxide. It should be mentioned that the absence of metallic Pd (111) peak at 2θ = 40° (Pd, JCPDS 05-0681), could be explained by the high dispersion of metal particles that escape the XRD detection; TEM analysis would assist on that front [37].

### 3.3. Textural Analysis

**Figure 3:**
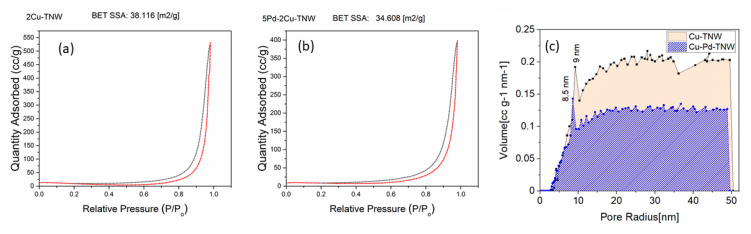
N_2_ adsorption–desorption isotherms over (**a**) Cu-TNW; (**b**) Pd-Cu-TNW catalysts; (**c**) BJH pore size distribution.

The N_2_ adsorption/desorption isotherms for selected mono- and bi-metallic catalysts, namely Cu-TNW and Pd-Cu-TNW are presented in Figure 3. Both catalysts show type IV isotherms and H3 hysteresis loop indicating mesoporous structure with pore width in the range of 2–50 nm according to IUPAC classification [33,40]. The hysteresis loop appearing in the multilayer range of physisorption isotherms is attributed to capillary condensation taking place in the mesopores [41]. Generally, it can be seen that there is no change in the shape of the isotherm for both catalysts, as we move from Cu to the Pd-Cu system, compared to TiO_2_ P25 isotherm previously reported in our study of CuO-TNT [19]. This indicates that the support maintained its pore structure upon Cu or Pd/Cu addition.

However, as expected, the BET surface area exhibited a slight decrease upon metal introduction. The further addition of Pd, led to a further decrease in the surface area. The surface area of Cu-TNW and Pd-Cu-TNW were found to be 38.1 and 34.6 m^2^/g, respectively (Table 1). This is an expected pattern and can be attributed to the pore filling by the added Cu^2+^ and Pd^2+^ which are incorporated and entrapped in the cavities of the support [9,12]. Similar decrease of BET surface area upon metal doping was reported by De Queiroz et al. and Wang et al. in their study of Cu/TiO_2_–Cu/Al_2_O_3_ catalysts and Pd/Cu/MOx (MOx=TiO_2_ and Al_2_O_3_) catalysts, respectively [9,40]. Hossain et al. also shows that both pore volume and surface area decreased upon incorporation of Cu in the titanium nanotube structure [29]. BJH size distribution in (Figure 3c) shows that Cu-TNW and Pd-Cu/TNW poses major size peak at around 9 nm with 0.83 and 0.62 cc/g total pore volume, respectively.

### 3.4. Surface Properties

**Figure 4:**
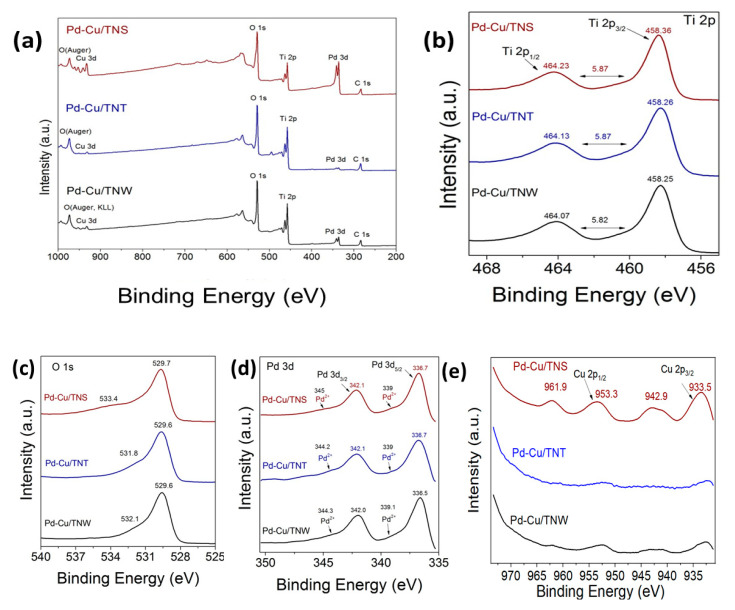
(**a**) Survey XPS spectra obtained over the Pd-Cu/TNS, Pd-Cu/TNT, and Pd-Cu/TNW catalysts; high-resolution XPS spectra over Pd-Cu/TNS, Pd-Cu/TNT, and Pd-Cu/TNW catalysts; (**b**) Ti 2p; (**c**) O 1s, (**d**) Pd 3d, and (**e**) Cu 2p core level spectra.

XPS was used to further investigate the chemical environment at the surface of the catalysts and understand the oxidation state of the different elements. The XPS core level (high resolution, HR) spectra of Ti 2p, O 1s, Pd 3d, and Cu 2p are shown in Figure 4 for Cu-Pd/TNS, Cu-Pd/TNT, and Cu-Pd/TNW. The HR-XPS scan for the Ti 2p in the different TiO_2_ was acquired from 450 to 477 eV. For Cu 2p and O 1s, the HR-XPS scan was acquired from 925 to 980 eV and from 525 to 540 eV, respectively. While for Pd 3d, the scan was collected from 335 to 350 eV. The binding energy of the peaks can be found in Table S7.

High resolution spectrum of Ti 2p region of Pd-Cu/TiO_2_ (Figure 4b), revealed two peaks at 458.25 eV in TNW and 458.26 eV in TNT and a second peak at 464.07, 464.13 for TNW and TNT support system, respectively. Similarly, Pd-Cu/TNS shows two peaks at 458.36 and 464.23 eV. Those two peaks are assigned to Ti 2p_3/2_ and Ti 2p_1/2_ at the higher binding energy side. A very slight shift can be observed especially in TNW- and TNT-based catalyst compared to TiO_2_ alone. The observed shift of the order of 0.04/0.05 eV toward lower binding energy (BE) can be attributed to oxygen vacancy generation and Ti^3+^ formation upon reduction due to the presence of copper. This structural disorder due to Cu addition can also be confirmed by the XRD studies (Figure 2B) and the shape change of the (101) peak of anatase. The down shift in Ti 2p peaks was also reported by Cheng-Yen Tsai and Deng et al. in their study of Cu-doped TiO_2_ and CuO supported on CeO_2_-doped TiO_2_, respectively [42,43] and shows similar trend with our previous work [19]. In addition, it can be clearly seen that the distance between the main Ti 2p_3/2_ and Ti 2p_1/2_ is about 5.87 eV in all of the different-shaped TiO_2_-based catalysts, indicating that TiO_2_ structure of the support is preserved upon the addition of metals [19].

**Figure 5:**
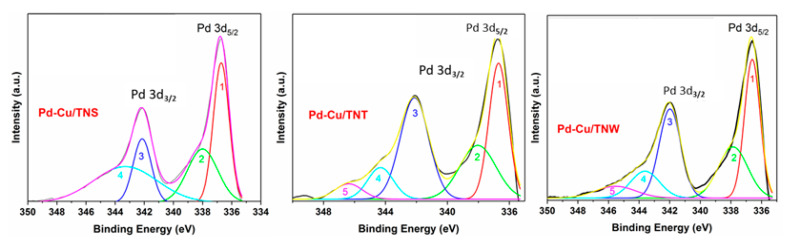
Peak deconvolution for Pd 3d for Pd-Cu/TNS, Pd-Cu/TNT, and Pd-Cu/TNW.

The high resolution XPS spectra of Pd (3d) core level of the three Pd-Cu/ TiO_2_ catalysts presented in Figure 4d, are featured with two (2) main peaks centered at around 337.1 and 342.5 eV, that can be attributed to metallic Pd in 3d_5/2_ and 3d_3/2_ regions, while the other two peaks observed at around 338.3 and 343.7 eV, are assigned to Pd^2+^ species most likely in PdO (in agreement with the XRD findings) [11]. The overlapped peaks were fitted by Gaussian curves [11]. The details of the peak fitting analysis can be found in the Supplementary Tables S1–S3 and Figure 5. In the Pd 3d_5/2_ and 3d_3/2_ regions, a clear positive shift toward higher binding energy was observed in all the prepared catalysts of this study. Compared to Pd binding energy reported in literature [44], around 1.3 eV upshift demonstrate the strong Pd interaction with the TiO_2_ support. This is similar to what have been observed in other studies of Pd incorporation [11,12,45,46]. Furthermore, the formation of Cu-Pd alloy can enhance the shifting of the binding energy. The shift in the binding energy of Pd in bimetallic system compared to monometallic was explained by the induced modification in the electronic structure of the valence d band [46].

As can be seen in Figure 4e, high resolution XPS spectrum of the Cu(2p) core level region of the Cu-Pd/TiO_2_ indicate Cu 2p_3/2_ and Cu 2p_1/2_ core peaks. The peak deconvolutions revealed the presence of four (4) main peaks. The peak located at ~933.5 eV corresponds to Cu^2+^, while strong peaks at around 942.9 and 961.9 eV can be assigned to Cu^2+^ (CuO phase). The other shakeup satellite peaks at 950–960 eV and 940–945 eV, can be attributed to the presence of unfilled 3d or multielectron excitation as reported in other studies of Cu [12,47,48] and it can be used as an indication of Cu^2+^ presence [48,49]. The peak expected at 953 eV combined with the weak satellite peak indicate the presence of Cu^+^. Overall, it can be observed that Cu 2p peaks in all Cu-Pd/TiO_2_ catalysts shifted to higher binding energy in comparisons to CuO alone [19]. The presence of the support, lead to peak shifting to higher binding energy which can reflect the effect of strong metal- support interaction at the catalyst interface. The location of the Cu 2p_3/2_ and Cu 2p_1/2_ binding energy are comparable to other similar catalysts prepared by Yahia H. Ahmad and Wenliang Gao et al. [12,50] and indicate the presence of both Cu^2+^ and Cu^+^.

However, it should be noted that this upshift might be counteracted to some extent by the palladium addition. Chiba et al. in their study of Cu-Pd nanoparticles reported as the palladium ratio increased, Cu 2p _3/2_, shifts to lower binding energy [46]. The downshift was explained based on the Cu-Pd alloy formation, which may cause electronic structural change for the valance d bands of Cu and Pd. The signal in the TNT and TNW are much lower than the TNS-based catalysts and hardly distinguished from the background, this may be due to the well incorporation of the Cu inside the morphology of titanium wire and titanium nanotube which are formed by rolling up the two-dimensional sheets of TiO_2_ structure.

**Figure 6:**
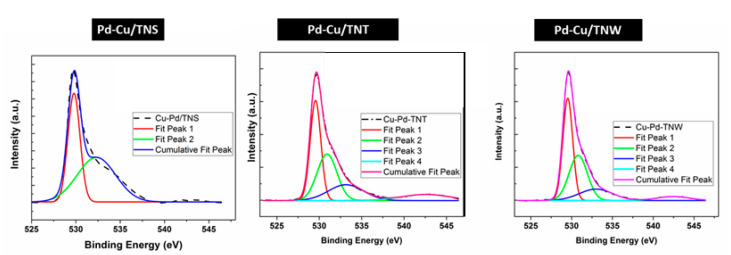
Peak deconvolution for O1s XPS for Pd-Cu/TNS, Pd-Cu/TNT, and Pd -Cu/TNW.

The O1s overlapped peaks are resolved into two (2) distinct peaks at around 532 eV and 530.2 eV for all catalyst compositions (Figure 4c). The lower binding energy peak corresponds to the presence of lattice oxygen species [27,33,51] while the peak at around 532 reveals the presence of adsorbed oxygen in hydroxyl species that is present on the surface. Moreover, it can be attributed to carbonate or polarized O^2−^ ions located adjacent to O vacancy sites or to the oxygen in CuO [27,33,51]. According to the literature, the O 1s binding energy of metal hydroxides and metal carbonates are typically around 531.0–531.5 eV [51]. Generally, the peak at the higher binding energy sites only appear in the bimetallic catalysts. This confirms the role of Cu-Pd addition in introducing modification to the oxygen vacancy sites which can play a key role in enhancing CO-oxidation. Furthermore, it can be seen that Pd addition leads to a further upshift of the shoulder peak to a higher binding energy compared to Cu-TNT alone. In our previous study, the O1s peak shifts from 531.5 eV in CuO-TNT to 532.2 in Pd-Cu/TNT in the current study [19]. Similar shift to higher binding energy can be found using TNS support (533.8 eV) and TNW support (532.5 eV). Deconvolution of the O1s peak has been performed for better understanding of the oxygen components (see Figure 6 and Tables S4–S6).

### 3.5. Vibrational Studies

**Figure 7:**
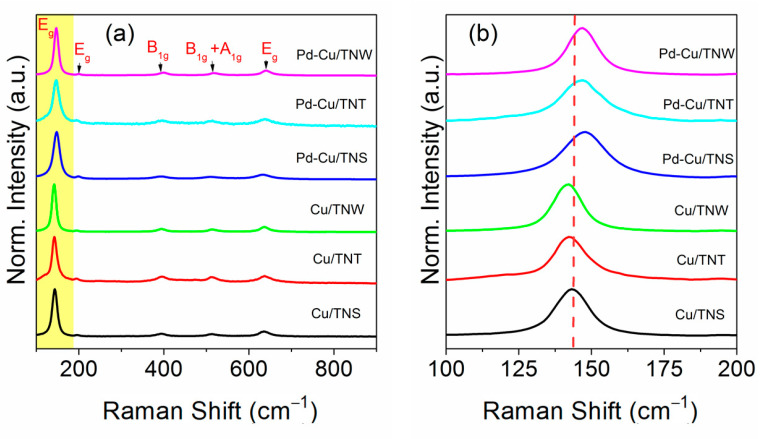
(**a**) Raman spectra of Cu-TNS, Cu-TNT, Cu-TNW, Pd-Cu-TNS, Pd-Cu/TNT, Pd-Cu/TNW catalysts (**b**) Raman spectra of Eg mode of TiO_2_.

Figure 7 represents the Raman spectra of the Pd-Cu/TiO_2_ nanostructure system synthesized on three different support morphologies namely nanosphere (NS), nanotube (NT), and nanowire (NW) titanium oxide. In addition, the Raman spectra of the pure Cu catalyst on the same supports’ morphologies (monometallic systems) were added for comparison. Raman spectra is an important probe tool for the M-O bond environment and the structural changes as those are reflected in the characteristic bands [25,27]. As shown in Figure 7, all the Raman bands are associated with TiO_2_ anatase phase. The six active Raman modes of anatase phase are identified as 3E_g_ modes, 1A_1g_ mode, and 2B_1g_ modes that are attributed to symmetric stretching vibration, antisymmetric stretching vibration, and bending vibration of O-Ti-O, respectively [18,19]. In all the cases of herein catalysts, there is a shift of the position of the main E_2g_ peak to higher wavenumber accompanied by peak broadness. Compared to TiO_2_ Eg peak that was reported in our previous study and in the literature, the peak upshifts from 140 cm^−1^ to 143.2 or 142.2 cm^−1^ upon addition of Cu to the support [19,52,53]. The shift becomes more pronounced upon adding the second metal, Pd, to the structure, as the vibrational mode shifts to 147.1 cm^−1^ for Pd-Cu/TNT, Pd-Cu/TNW and to 148.0 cm^−1^ for Pd-Cu/TNS. It is well-known that addition of metal to the support will induce structural change and defects in the crystal. Due to the difference in size of copper ion (Cu^2+^, 0.7 Å) or palladium ion (Pd^2+^, 0.7 Å) and Ti^4+^ ions (0.6 Å), TiO_2_ lattice structure is anticipated to be distorted following Cu/Pd addition [54]. Furthermore, the charge difference between the copper/palladium and the titanium ion on the substitutional sites will contribute to the structural distortion and lead to oxygen vacancies formation in the titanium oxide lattice [52,55]. It can be concluded that all the observed upshift in the Raman main E_g_ peak, can be related to the presence of oxygen vacancies induced by Cu-Pd addition. The presence of oxygen vacancies and surface defects in our study was also confirmed using XPS results (see above Figure 6). According to K. Polychronopoulou et al. phonon confinement and lattice strain upon doping metal on substrate ions can also affect Raman active modes leading to oxygen vacancies generation [28]. The other four important characteristic peaks in the Raman spectra of the prepared catalyst are positioned at 194 cm^−1^ (Eg mode), ~394 cm^−1^ (B_1g_ mode) TiO_2_ anatase phase, 514 cm^−1^ that is associated with B_1g_+A_1g_ vibrational mode, and 636 cm^−1^ that results from Eg mode [19,56]. These peaks positions were not largely influenced by the addition of the Cu/Pd metals to the support. It is worth mentioning that there is a slightly higher positive shift upon addition of Cu-Pd metals into the titanium oxide in the case of TNW morphology. The peak shifts to 399.68 cm^−1^ (B_1g_) and 639.77 cm^−1^ (Eg) compared to the reported value of TiO_2_ support alone (at 393 and 636 cm^−1^ respectively) [19,56]. This can clearly show that the morphology of the supports can play a role in the extend of the interaction between the metal and support. The nanowire may provide more exposed surface that can facilitate the degree of Cu-Pd interaction with the support surface.

Overall, it can be seen that the TiO_2_ supports upon Cu and Pd addition facilitates the oxygen vacant sites formation. Furthermore, the absence of any CuO or PdO peak can indicate the fine size of the metal/bimetal. However, it should be mentioned that contradiction between XRD and Raman results in term of PdO presence, could be attributed to the nature of Raman analysis as a surface characterization technique (Raman peak of PdO is masked by the one corresponding to TiO_2_). The oxygen vacancy creation upon metal addition in TiO_2_, has been reported in different studies and are in good agreement with our observations [18,30,52,57].

### 3.6. Catalytic Activity and Stability

**Figure 8:**
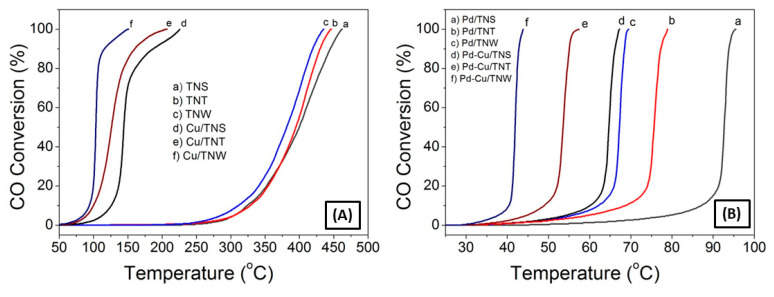
CO oxidation light-off curves of TNS, TNT and TNW support (**A**) before and after Cu loading to the support alone, (**B**) before and after adding Cu to Pd-TiO_2_ support.

The catalytic activity of the different catalysts toward the CO oxidation reaction was measured using a continuous flow fixed-bed catalytic reactor. Figure 8 displays the catalytic activity under the feed gas containing 4% CO and 20% O_2_ (with Ar as balance).

TiO_2_ bare supports have low CO oxidation activity. As shown in Figure 8A, the different shaped bare TiO_2_ nanoparticles exhibit a poor catalytic activity toward the oxidation of CO with no significant activity below 300 °C, where the CO oxidation light off curve reveals T_50_ of 399.8 °C, 394.7 °C, and 381.4 °C for TNS, TNT, and TNW, respectively (T_50_ is the corresponding reaction temperature when the conversion is 50%). Our prepared TiO_2_ nanotube showed a little bit lower activity compared to the TiO_2_ nanotube prepared by sol-gel and hydrothermal treatment methods, showing T_50_ of 210 °C [1].

When comparing the T_50_ values for the Cu-TiO_2_ catalysts, it can be seen that, the catalytic activity was significantly increased, with a T_50_ of 144.1 °C followed by T_50_ of 126.2 °C and 103.3 °C for Cu/TNS, Cu/TNT, and Cu/TNW, respectively. This can be attributed to the presence of readily reducible oxygen moieties adjacent to the Cu ion surface. In previous study [19], it was shown that the catalytic activity of CuO-TiO_2_ NT for CO oxidation is attributed to the interplay between the CuO and TiO_2_ counterparts and the strong metal–support interaction at the interface. It should be mentioned that Cu is not the only key factor; for the same Cu loading, different oxidation reactivity rates were observed due to the different shapes and microstructure of the supports. The highest catalytic activity was achieved using nanowire support with T_50_ of 103.4 °C and T_100_ of 150.2 °C. This is mostly due to the expected higher surface area along with the uniform distribution of the Cu ion on the TNW support. As a result, the nanowire morphology can lead to more exposed active sites and enhanced activity.

In the case of Pd-based catalyst, further increase in the CO conversion was observed, as reflected by the T_50_ of 92.7 °C, 75.6 °C, and 67.2 °C for the Pd/TNS, Pd/TNT, and Pd/TNW catalysts, respectively. The boost of the activity is reflected onto the T_50_ drop of almost 1.6 times compared to T_50_ of the support alone. This is most likely due to the presence of Pd ions which are widely known to enhance the catalytic activation of CO. Pd metal by itself is a famous CO-oxidation catalyst with high catalytic activity [6,37,58]. However, it was reported that the use of palladium metal alone without using a support will hinder the long-term stability of the catalyst due to metal sintering [7,9,59]. In addition, the high price of this noble metal hinders its use in large-scale application and motivates the study toward the use of support and combination with other cost-effective metals in order to reduce the amount required for such noble metals. Satsuma et al. reported the effect of loading Pd on different supports (CeO_2_, TiO_2_, Al_2_O_3_, ZrO_2_, and SiO_2_) for CO oxidation at low temperatures [60]. The highest CO oxidation was found using CeO_2_ or TiO_2_ as a support. A recent publication by the same group has reported a further improvement in the catalyst activity with the use of Pd catalyst supported on titania-coated ceria (T_100_ ~135 °C) [6]. On the other hand, Cheng Hao Wu and his team reported the use of Pd as bimetal system without a support. They reported that CoPd bimetallic nano-catalyst achieved a complete CO conversion at temperature as low as 110 °C and around 180 °C for Pd catalyst alone [58]. This is still higher temperature compared to our reported Pd on TiO_2_ (T_100_ = 69.5 °C for Pd/TNW and 95.4 °C in Pd/TNS).

Comparing the catalytic activity of the different catalysts studied in our works, it can be seen that the Pd-Cu catalyst supported on TiO_2_ exhibit the highest catalytic activity with T_50_ = 64.6 °C for Pd-Cu/TNS, T_50_ = 53.5 °C for Pd-Cu/TNT, and T_50_ = 41.9 °C for Pd-Cu/TNW, so the Pd catalysts modification by nanointerface with Cu and supporting it on TiO_2_ nanowires demonstrated the highest enhancement in CO oxidation rates with 100% CO conversion at temperature regime down to near-ambient temperatures (T_100_ = 43.85 °C). This result reflects clearly the synergetic effect between Cu, Pd and the TiO_2_ supports. In addition, a better dispersion of Cu and Pd on the optimized support morphology can provide more population of active site and hence greater CO surface coverage. Overall, it can be stated that the order of catalytic activity toward CO oxidation is as follows TiO_2_ < Cu/TiO_2_ < Pd/TiO_2_ < Pd-Cu/TiO_2_.

Numerous groups have reported on the CO oxidation activity using similar catalysts system (Table S8). For example, the bimetallic Pd–Cu nano-catalysts have been studied previously in CO oxidation, but using another support, Al_2_O_3_. Interestingly, the 100%CO conversion was achieved at higher temperature (200 °C compared to 43.9 °C in our system) [5]. Similarly, Santos et al. reported a range of CO oxidation capacity varying from T_50_ =187 to 413 °C (Pt > Pd ≥ Rh ≈ Ir ≥ Au≥ TiO_2_), depending on the preparation methods and the noble metal loaded on TiO_2_ support [61]. Wang et al., have reported that CuO-nanorods-decorated reduced graphene oxide (RGO) nano-catalysts showed total CO oxidation at 165 °C [62], while M. Manolata Devi et al. shows full conversion of CO at 75 °C using Pd-Pt on graphene catalyst [3]. The achieved results are comparable with our system considering that Cu is cheaper in cost and the synthesis method is simpler.

Comparing CO oxidation performance with other affordable yet active systems, such as mixed oxides that were reported in the literature might not be accurate due to the different applied reaction conditions, but generally it indicates that the prepared system in our study has lower T_50_ than different ceria-based oxides e.g., Ce_x_Sm_1-x_O_2_ catalysts which are known among the candidates to have great potential for CO oxidation. For example, K. Polychronopoulou reported in their study a minimum of T_50_ = 300 °C for different mixed oxide systems [28]. However, it is worth mentioning that a recent study by Alketbi et al., showed an enhancement in CO oxidation with increasing Cu dopant content (20 at. % Cu) in the Ce-La-Ox catalysts (T_50_ =162 °C). Similarly, a recent study by Akhoori et al. revealed an enhanced CO oxidation activity in samarium-copper co-doped ceria catalysts. The CO oxidation reaction was promoted at low temperature (140 °C) mainly by increasing the Cu content. These studies agree well with our observations of the role of Cu addition in CO oxidation catalytic enhancement [25]. Interestingly, our present catalyst exhibits higher catalytic activity compared to many catalysts in the open literature.

Many factors play a role in controlling the activity of CO oxidation at low temperature. Satsuma et al. indicated in his study of Pd-supported catalyst, that TiO_2_ can contribute to enhancing the catalyst activity in CO oxidation through the induced reduction–oxidation cycle of supported Pd [6]. In another study, it was found that the role of TiO_2_ support as an oxygen storage medium and its contribution in oxygen release and storage property, are important factors in enhancing the CO oxidation [60], while equally important is the reducibility degree of the PdO to Pd metal [60]. Mozer and his group reported that addition of Cu to monometallic supported Pd can enhance or suppress the CO oxidation activity depending on the support type [63]. Cu presence reduced the CO oxidation when introduced to Pt/Al_2_O_3_ support. This was due to increase in CO desorption rate, as concluded from CO-TPD studies. Also, Cu can block the Pt active site hindering their activity. On the other hand, Cu addition to Pt/Nb_2_O_5_ can enhance the CO oxidation activity through improving the metal–metal interaction and Cu contribution in providing additional pathway for O_2_ adsorption over the reducible Nb_2_O_5_ oxide support.

Recently an interesting finding was reported by Polychronopoulou et al. in their study of using different doped ceria oxides in CO oxidation application [64]. The systematic study demonstrated that heteroatoms substitution will induce lattice distortion but the difference in their activity, can be mainly correlated to the difference in oxygen lattice mobility and the degree of reducibility of the doped metal cation. It was found that Cu doping led to the highest CO oxidation activity due to the high lattice oxygen mobility, synergetic effect between Cu and CeO_2_ support, and highest charge compensation in Cu-doped system compared to other heteroatom CeO_2_ systems. It should be mentioned that even though CeO_2_ and TiO_2_ are two different supports however, the governing principle is that by adding different metals at varying loadings leads to the formation of different types of oxygen vacancies (e.g., surface, sub-surface, single, clusters); the latter is what is suggested as a potential scenario in the herein case where Pd-Cu were co-added onto TiO_2_. Based on the XRD and Raman findings above, a structural distortion into the TiO_2_ lattice occurred most likely due to some incorporation of Cu into titania [Cu-O-Ti^4/3^] in the interface and possible Ov formation. This finding is highly correlated to the activity of the catalysts in CO oxidation, and can be expanded to the exploration of different heteroatom-doped system including TiO_2_.

**Figure 9:**
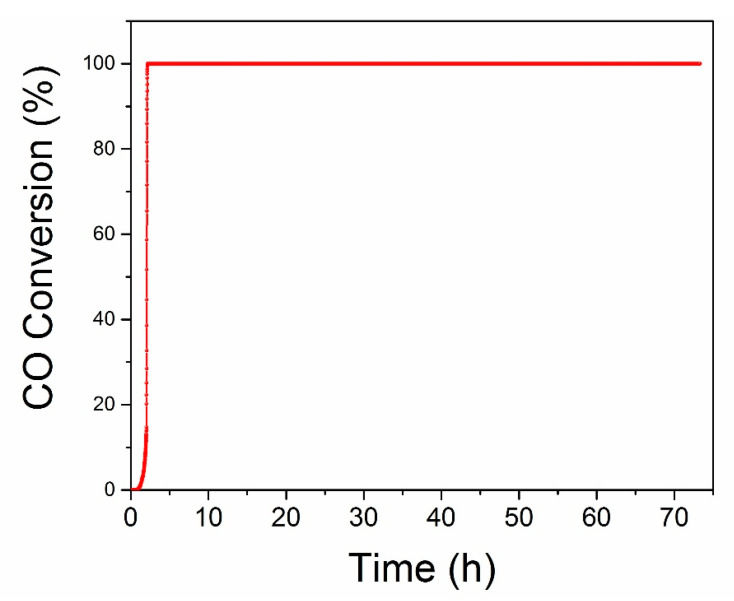
CO oxidation long term stability test of Pd-Cu/TNW under continuous stream for 72 h at ~60 ± 5 °C.

The long-term stability of Pd-Cu/TNW catalyst was evaluated under continuous stream for 72 h as shown in Figure 9. After passing the feed gas mixture over the catalyst, the temperature was increased and maintained at 60 ± 5 °C throughout the whole stability test for CO oxidation. The 100% conversion was rapidly achieved and the performance of the catalyst was continuously maintained during the whole period of study. This indicate that the catalyst is stable and was not subjected to deactivation within the time studied herein. The excellent activity and stability of the Pd-Cu/TNW catalyst enhances the practical value of the catalyst in real-life application.

### 3.7. Mechanistic Aspects of the CO Oxidation Reaction

Different catalytic CO reaction pathways have been proposed in previous studies to explain the catalytic activity toward CO oxidation. Among the proposed mechanisms, Langmuir–Hinshelwood [4,5,65], oxygen spillover [65,66], and Mars van Krevelen mechanisms are suggested as the most popular ones [26,60]. Langmuir–Hinshelwood mechanism was proposed in our previous study as a potential reaction for CO-oxidation, using CuO-TiO_2_ nanotube catalyst [19]. This mechanism suggests that both CO and O_2_ molecules are adsorbed on the surface. The CO adsorbs on the metal surface while O_2_ adsorbs on the support followed by O_2_ dissociation into atomic O. Eventually, the oxidation of the adsorbed CO proceeds through the reaction with the adsorbed O atom. This mechanism was reported recently by Wasim et al. as the leading mechanism in Cu-titanium nanorod catalysts [67]. Several studies in the open literature proposed Langmuir–Hinshelwood mechanism for Cu and Pd supported catalysts as well [19,68]. Fagen Wang proposed that this is mainly applicable for Cu-Pd on Al_2_O_3_, while more complex mechanisms are expected for TiO_2_ [9]. Atsushi Satsuma reported in his study of CO-oxidation over supported Pd catalyst, that titanium oxide contributes to oxygen supply and hence Mars-van Krevelen can be proposed as a more representative mechanism [60]. Mars-van Krevelen is the most prominent mechanism for CO oxidation over different catalysts, such as Cu/Pd-supported catalysts and ceria-based catalysts [24,26,60].

It is well-known that Ti^3+^ formation on the reducible TiO_2_ contributes to the formation of the oxygen vacant sites [8,69]. We suggest in our study that CO-oxidation proceeds on Pd-Cu/TiO_2_ catalyst by Mars-van Krevelen mechanism. The process has five main steps: (1) Initially, CO molecule chemisorbed on the active metal surface forming Pd-CO; (2) O_2_ gas-phase molecule binds at the interface between the support and the active metal surface, in our case O_2_ molecule binding to Cu^2+^/Cu^+^ active site was suggested. This step is followed by the dissociation of O_2_- and diffusion to the adjacent PdO site [12]; (3) the oxidation of CO by the lattice oxygen at the TiO_2_ and Pd-Cu alloy interface leads to the formation of oxygen vacant sites and subsequent reduction of Pd^2+^ to Pd^0^; (4) regeneration of the catalysts and continuity of the cycle is proposed to happen through oxygen vacancies interaction with the dissociated O_2_ gas-phase molecule; (5) the last step of the mechanism according to Thang and his group, involves oxidation of another CO molecule through the reaction with the extra O atom formed in step 4 [70].

**Figure 10:**
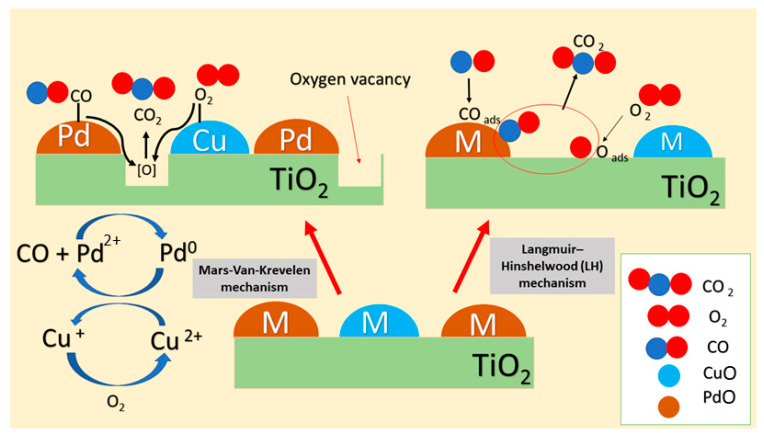
Proposed CO oxidation pathways over Pd-Cu/TiO_2_ catalyst.

Zhang et al. shows that the strong support interaction and the bifunctional mechanism contribute to the enhanced catalytic activity of Au-Cu supported on CeO_2_ [65]. Particularly in the case of bimetal alloy, it was shown that synergistic effect and CO-adsorption-induced surface aggregation are the known phenomena that play a key role in the catalytic activity determination [58,71,72]. In our case, it is believed that Cu will surface segregate and bind to the O_2_ gas molecule while Pd will mainly bind to the CO. The reoxidation of the Pd^0^ species by Cu^2+^ reduction will ensure the cycle is completed. After that Cu can be easily oxidized using O_2_ gas phase molecule. In general, the competition between the CO and O_2_ reactants for the binding sites is believed to contribute to the enhanced catalytic activity of the Cu-Pd bimetal supported on the different titanium oxide morphology [12,65]. Proposed CO oxidation pathways over Pd-Cu/TiO_2_ catalyst are demonstrated in Figure 10, but in order to verify the mechanism for the promoted activity, more experiments such as in situ DRIFTS studies should be conducted.

## 4. Conclusions

In this work, we have demonstrated the promoting effect of the TiO_2_ support morphologies and the Pd-Cu nano interfacing on the CO oxidation reaction over Pd-Cu/TiO_2_ catalysts. It was found that the synergy effect between the Cu-Pd and titanium oxide nanowire leads to the most enhanced catalytic activity at lower temperature close to near-ambient conditions. Pd-Cu-based catalysts were developed and supported on three (3) different TiO_2_ morphology namely nanosphere, nanotube, and nanowire. The results revealed that CO oxidation performance is in the following order TiO_2_ < Cu/TiO_2_ < Pd/TiO_2_ < Pd-Cu/TiO_2_. It was suggested that the alloying between Cu and Pd metal, facilitate their dispersion in the support. Moreover, nanowire provides more exposed surface with reducible oxygen species and a different anchoring of the Pd, Cu species on it. This led to a considerable improvement in catalytic activity and stability to below 100 °C (T_100_ = 44 °C) using Pd-Cu^/^TiO_2_ nanowire. The enhanced interaction between the CO and the oxygen species on the defects sites was correlated and investigated by using different analysis including XRD, Raman, XPS, SEM, and BET physisorption. The development of active oxygen sites was reflected by the up-shift of the E_2g_ mode of TiO_2_ in the Raman spectrum and the lower binding energy shift of the Ti 2p_3/2_ XPS peak. The understanding presented herein can be extended to tuning other metal–metal oxides interfaces used in the CO oxidation reaction.

## Figures and Tables

**Table 1:** Textural properties along with crystallite size of different prepared catalysts of this study.

Catalyst	Crystal Size (nm) *	Surface Area (m^2^/g)	Pore Volume (cm^3^/g)
Cu-TNS	29.5	-	-
Cu-TNT	9.2	-	-
Cu-TNW	14.1	38.1	0.83
Pd-Cu/TNS	27.6	-	-
Pd-Cu/TNT	13.9	-	-
Pd-Cu/TNW	32.1	34.6	0.62

* by applying Scherrer equation on the measured from TiO_2_ (101) peak based on Scherrer formula.

## Data Availability

Data can be provided upon request.

## Supplementary Materials

The following are available online at https://www.mdpi.com/article/10.3390/nano11071675/s1, Table S1: Gaussian fit and Peak deconvolution data for Pd 3d XPS in Pd-Cu/TNS, Table S2: Gaussian fit and Peak deconvolution data for Pd 3d XPS in Pd-Cu/TNT, Table S3: Gaussian fit and Peak deconvolution data for Pd 3d XPS in Pd-Cu/TNW, Table S4: Gauss fit and Peak deconvolution data for O1s XPS in Pd-Cu/TNS, Table S5: Gauss fit and Peak deconvolution data for O1s XPS in Pd-Cu/TNT, Table S6: Gauss fit and Peak deconvolution data for O1s XPS in Pd-Cu/TNW, Table S7: XPS peak binding energy assignments, Table S8: CO oxidation activity of our catalysts and other related catalysts.
