# Supplementary material for: CO Oxidation at Near-Ambient Temperatures over TiO2-Supported Pd-Cu Catalysts: Promoting Effect of Pd-Cu Nanointerface and TiO2 Morphology
Source: Nanomaterials (Basel). 2021 Jun 25;11(7):1675. doi: 10.3390/nano11071675 (PMC8306827; doi:10.3390/nano11071675)
Supplement: Supplementary file 1 [file nanomaterials-11-01675-s001.zip › nanomaterials-1180822-supplementary.pdf]

nanomaterials

MDPI

Supplementary Material

CO Oxidation at Near-Ambient Temperatures over TiO_2-Supported Pd-Cu Catalysts: Promoting Effect of Pd-Cu Nanointerface and TiO_2 Morphology

Abdallah F. Zedan [1,*], Safa Gaber [2], Amina S. AlJaber [3] and Kyriaki Polychronopoulou [2,4,*]

[1] National Institute of Laser Enhanced Science, Cairo University, Main Campus, Giza 12613, Egypt
[2] Center for Catalysis and Separations, Khalifa University of Science and Technology, Abu Dhabi P.O. Box 127788, United Arab Emirates; saagaber90@gmail.com
[3] Department of Chemistry, Qatar University, Doha 2713, Qatar; a.s.aljaber@qu.edu.qa
[4] Department of Mechanical Engineering, Khalifa University of Science and Technology, Abu Dhabi P.O. Box 127788, United Arab Emirates
* Correspondence: azedan@niles.edu.eg (A.F.Z.); kyriaki.polychrono @ku.ac.ae (K.P.); Tel.: +2-02-35675236 (A.F.Z.)

Table S1. Gaussian fit and Peak deconvolution data for Pd 3d XPS in Pd-Cu/TNS.

Model	Gaussian			
Equation	y = y0 + A/(w*sqrt(pi/(4*ln(2)))) * exp(−4*ln(2)*(x-xc)^2/w^2)			
Plot	Peak1(B)	Peak2(B)	Peak3(B)	Peak4(B)
y0	−0.00562 ± 0.0033	−0.00562 ± 0.0033	−0.00562 ± 0.0033	−0.00562 ± 0.0033
xc	336.71876 ± 0.00825	337.99862 ± 0.15579	342.14296 ± 0.01379	343.29934 ± 0.11648
A	1.01823 ± 0.10132	0.74188 ± 0.12277	0.5292 ± 0.02864	1.05198 ± 0.05865
w	1.28561 ± 0.04191	2.44695 ± 0.26283	1.46628 ± 0.04586	5.16122 ± 0.18801
Reduced Chi-Sqr	$1.76019*10^{-4}$			
R-Square (COD)	**0.99655**			
Adj. R-Square	0.99626			

Table S2. Gaussian fit and Peak deconvolution data for Pd 3d XPS in Pd-Cu/TNT.

Model	Gaussian				
Equation	y = y0 + A/(w*sqrt(pi/(4*ln(2)))) * exp(−4*ln(2)*(x-xc)^2/w^2)				
Plot	Peak1(E)	Peak2(E)	Peak3(E)	Peak4(E)	Peak5(E)
y0	0.68289 ± 0.00192	0.68289 ± 0.00192	0.68289 ± 0.00192	0.68289 ± 0.00192	0.68289 ± 0.00192
xc	336.69738 ± 0.01318	338.03611 ± 0.33166	342.12392 ± 0.05905	344.32183 ± 0.1558	346.37533 ± 0.43852
A	0.41585 ± 0.07728	0.31292 ± 0.0873	0.46595 ± 0.03369	0.12966 ± 0.05476	0.06757 ± 0.03534
w	1.44888 ± 0.08567	2.75708 ± 0.46016	2.17613 ± 0.11676	1.94451 ± 0.55714	2.08056 ± 0.66893
Reduced Chi-Sqr	$6.22459*10^{-5}$				
R-Square (COD)	**0.99226**				
Adj. R-Square	0.99141				

Table S3. Gaussian fit and Peak deconvolution data for Pd 3d XPS in Pd-Cu/TNW.

Model	**Gaussian**				
Equation	y = y0 + A/(w*sqrt(pi/(4*ln(2)))) * exp(−4*ln(2)*(x-xc)^2/w^2)				
Plot	Peak1(C)	Peak2(C)	Peak3(C)	Peak4(C)	Peak5(C)
y0	0.37871 ± 0.00555	0.37871 ± 0.00555	0.37871 ± 0.00555	0.37871 ± 0.00555	0.37871 ± 0.00555
xc	336.60336 ± 0.01681	337.86171 ± 0.23142	341.97893 ± 0.12538	343.61177 ± 0.46675	345.53395 ± 3.48824
A	0.70187 ± 0.09827	0.48683 ± 0.11289	0.62606 ± 0.20328	0.25045 ± 0.56937	0.15445 ± 0.42512
w	1.20458 ± 0.05793	2.26087 ± 0.34057	1.67245 ± 0.1387	2.21717 ± 2.09567	3.11643 ± 3.69335
Reduced Chi-Sqr	$3.36527*10^{-4}$				
R-Square (COD)	**0.9882**				
Adj. R-Square	0.9869				

Table S4. Gauss fit and Peak deconvolution data for O1s XPS in Pd-Cu/TNS.

Model	**Gauss**	
Equation	y=y0 + (A/(w*sqrt (PI/2)))*exp(−2*((x-xc)/w)^2)	
Plot	Peak1(B)	Peak2(B)
y0	0.00435 ± 0.00211	0.00435 ± 0.00211
xc	529.78156 ± 0.01034	532.31517 ± 0.09681
w	1.36817 ± 0.03138	4.33284 ± 0.15758
A	1.07753 ± 0.03993	1.40001 ± 0.06271
Reduced Chi-Sqr	$4.74279*10^{-4}$	
R-Square(COD)	**0.98608**	
Adj. R-Square	0.98571	

Table S5. Gauss fit and Peak deconvolution data for O1s XPS in Pd-Cu/TNT.

	**Gauss**			
Equation	y=y0 + (A/(w*sqrt(PI/2)))*exp(−2*((x-xc)/w)^2)			
Plot	Peak1(B)	Peak2(B)	Peak3(B)	Peak4(B)
y0	$-0.00235 \pm 8.8233*10^{-4}$	$-0.00235 \pm 8.8233*10^{-4}$	$-0.00235 \pm 8.8233*10^{-4}$	$-0.00235 \pm 8.8233*10^{-4}$
xc	529.56114 ± 0.00445	530.91783 ± 0.07997	533.14957 ± 0.53227	542.57941 ± 0.08813
w	1.2495 ± 0.01998	2.32688 ± 0.18827	4.14618 ± 0.47508	5.66583 ± 0.24001
A	0.95349 ± 0.05547	0.82529 ± 0.17062	0.50057 ± 0.13066	0.26406 ± 0.0119
Reduced Chi-Sqr	$2.24717*10^{-5}$			
R-Square(COD)	**0.99935**			
Adj. R-Square	0.99932			

Table S6. Gauss fit and Peak deconvolution data for O1s XPS in Pd-Cu/TNW.

Model	**Gauss**			
Equation	y=y0 + (A/(w*sqrt(PI/2)))*exp(−2*((x-xc)/w)^2)			
Plot	Peak1(B)	Peak2(B)	Peak3(B)	Peak4(B)
y0	$-0.00204 \pm 8.56101*10^{-4}$	$-0.00204 \pm 8.56101*10^{-4}$	$-0.00204 \pm 8.56101*10^{-4}$	$-0.00204 \pm 8.56101*10^{-4}$
xc	529.52408 ± 0.0039	530.80478 ± 0.0785	532.9962 ± 0.58344	542.45557 ± 0.09058
w	1.20555 ± 0.01807	2.23169 ± 0.16432	4.10906 ± 0.52062	3.97714 ± 0.22041
A	0.94107 ± 0.05114	0.7681 ± 0.1393	0.3573 ± 0.10215	0.12177 ± 0.00876
Reduced Chi-Sqr	$2.0319*10^{-5}$			
R-Square(COD)	**0.9994**			
Adj. R-Square	0.99937			

Table S7. XPS peak binding energy assignments.

Species /core level	Peak	BE (eV) in Pd-Cu/TNS	BE (eV) in Pd-Cu/TNT	BE (eV) in Pd-Cu/TNW	Oxidation state
Ti 2p	Ti $2p_{3/2}$	458.36	458.26	458.25	
	Ti $2p_{1/2}$	464.23	464.13	464.07	
O 1s		530.1	530	530	
		533.8	532.2	532.5	
Pd 3d	Pd $3d_{5/2}$	337.1	337.1	336.9	Pd^0
	Pd $3d_{3/2}$	342.5	342.5	342.4	
		338.3	338.4	338.3	Pd^{2+}
		343.7	344.7	344	
Cu 2p	Cu $2p_{3/2}$	933.9	932.8	933.2	Cu^{2+} /Cu^{1+}
	Cu $2p_{1/2}$	953.7	952.6	953.2	
	satellite peak	943.3	942.8	943.1	
	satellite peak	962.3	962.1	962.7	

Table S8. CO oxidation activity of our catalysts and other related catalysts.

Catalyst	Synthesis method	T100 (°C)	T50 (°C)	Composition of the feed mixture	Space velocity mL^{-1} g^{-1} h^{-1})	Reference
**Pd/TiO_2-CeO_2**	impregnation method	~175 (T80 = 138)	~80	0.45% CO/10% O_2/ N_2	GHSV = 480 000 hr^{-1}	[1]
**Pd-Cu/Al_2O_3**	impregnation method	140	132	2.5% CO and 20% O_2 in N_2 balance	GHSV = 36,000 hr^{-1}	[2]
**Pd/Al_2O_3**	impregnation method	260	180	2.5% CO and 20% O_2 in N_2 balance	GHSV = 36,000 hr^{-1}	[2]
**Cu/Al_2O_3**	impregnation method	280	150	2.5% CO and 20% O_2 in N_2 balance	GHSV = 36,000 hr^{-1}	[2]
**50CuO-TiO_2 NT**	impregnation method	155	-	4% CO and 20% O_2 in a He balance	72,000	[3]
**Pd nanoparticle**	hydrothermal reduction	180	-	1.0% CO, 4.0% O_2 in He balance	60,000	[4]
**$Co_{0.24}$ $Pd_{0.76}$ bimetallic**	hydrothermal co-reduction	110	-	1.0% CO, 4.0% O_2 in He balance	60,000	[4]
**$Cu/Pd/Al_2O_3$**	deposition–precipitation and impregnation	~224.9	~124.9	2%CO + 1% O_2+ 97% He	46 700	[5]
**Pt–Cu/Nb_2O_5**	incipient wetness technique	-	150	30% H_2; 1%CO; 1% O_2 ; 20%CO_2; 10% H_2O; He balance	-	[6]
**Pt–Cu/Al_2O_3**	incipient wetness technique	-	350	30% H_2; 1%CO; 1% O_2; 20%CO_2; 10% H_2O; He balance	-	[6]
**20%Cu/Al_2O_3**	impregnation method	~540	~420	1600 ppm CO, 1.9% O2 in He	-	[7]
**2Ce–20Cu/Al_2O_3**	impregnation method	440	~250	1600 ppm CO, 1.9% O2 in He	-	[7]
**10.5La_2O_3–20CuO/Al_2O_3**	impregnation method		250	1600 ppm CO, 1.9% O_2 in He	-	[7]
**graphene-PdPt alloy nanoparticles**	wet-chemical route	158 (nanoflower)	-	CO (0.982%) and O_2 (0.493%) in H_2 flow at	GHSV = 47746 hr^{-1}	[8]
		75 (urchin)	-			
**Ce-La-20Cu-O**	microwave radiation coupled with sol-gel method	225	160	4% CO, 20% O_2 and balance He	60,000	[9]
**Pt, Pd, Ir, Rh and Au) supported on TiO_2**	liquid phase reduction deposition	165 (Au=Pt) > Pd > Rh > Ir 290	-	5% CO, 10% O_2 in He	-	[10]
**Pd supported on (CeO_2 ,TiO_2 ,Al_2O_3 ,ZrO_2 and SiO_2**	impregnation method	150 in Pd/CeO_2 < Pd/TiO_2 < Pd/Al_2O_3 < Pd/ZrO_2 ≤ Pd/SiO_2	97 Pd/CeO and 168 in Pd/SiO_2	0.45% CO/10% O_2/He	GHSV = 480,000 hr^{-1}	[11]

**Pd_{48}-Cu_{52}/Ce_2O_3**	wet chemical synthesis	-	~110	0.5 vol % CO + 10 vol % O_2balanced by N_2	GHSV = 16,000 hr^{-1}	[12]
**Pd_{48}-Cu_{52}/TiO_2**	wet chemical synthesis	-	70	0.5 vol % CO + 10 vol % O_2balanced by N_2	GHSV = 16,000 hr^{-1}	[12]
**Pd/(Ni,Co,Cu)/HNTs**	impregnation method	119–146	122–164	4 vol% CO, 20 vol% O_2	72,000	[13]
**AuCu/TiO_2**	solvothermal	125	80	1 vol% CO, 5 vol% O_2	20000	[14]
**CuO/TiO_2**	co-precipitation	120	76	1% CO + 5% O_2 (balance N_2)	60 000	[15]
**CuO/TiO_2**	impregnation	T90=188	-	1% CO + 5% O_2 (balance N_2)	60 000	[15]
**CuO-NPs/50-facet Cu_2O**	thermal using ascorbic acid as a reducing agen	~175		% $O_2$and 4% CO in He	-	[16]
**Cu-TNS**	wet impregnation	225.8	144.1	4%CO and 20% O_2 (with Ar as balance	72,000	
**Cu-TNT**	wet impregnation	205.1	126.2	4%CO and 20% O_2 (with Ar as balance	72,000	
**Cu-TNW**	wet impregnation	148.7	103.3	4%CO and 20% O_2 (with Ar as balance	72,000	
**Pd-TNS**	wet impregnation	95.4	92.7	4%CO and 20% O_2 (with Ar as balance	72,000	
**Pd-TNT**	wet impregnation	78.9	75.7	4%CO and 20% O_2 (with Ar as balance	72,000	This work
**Pd-TNW**	wet impregnation	69.5	67.2	4%CO and 20% O_2 (with Ar as balance	72,000	
**Pd-Cu/TNS**	wet impregnation	67.2	64.6	4%CO and 20% O_2 (with Ar as balance	72,000	
**Pd-Cu/TNT**	wet impregnation	57.4	53.5	4%CO and 20% O_2 (with Ar as balance	72,000	
**Pd-Cu/TNW**	wet impregnation	43.8	41.92	4%CO and 20% O_2 (with Ar as balance	72,000	
**(Cu, Co, Mn, Fe, Ni, Zr, and Zn)/CeO_2**	microwave assisted sol−gel synthesis	~575(Zn) – 75 (Cu)	~(275) Zn> Fe> Mn> Ni> Co> Cu (63)	4 vol% CO, 20 vol% O_2 (He as balance gas)	60,000	[17]

References

1. Satsuma, A.; Yanagihara, M.; Osaki, K.; Saeki, Y.; Liu, H.; Yamamoto, Y.; Arai, S.; Ohyama, J. Promotion of low-temperature oxidation of CO over Pd supported on titania-coated ceria. *RSC Adv.* **2014**, *4*, 54187–54193, doi:10.1039/c4ra10167g.
2. Wang, F.; Lu, G. Hydrogen feed gas purification over bimetallic Cu–Pd catalysts – Effects of copper precursors on CO oxidation. *Int. J. Hydrogen Energy* **2010**, *35*, 7253–7260, doi:10.1016/j.ijhydene.2009.12.186.
3. Zedan, A.F.; Allam, N.K.; AlQaradawi, S.Y. A Study of Low-Temperature CO Oxidation over Mesoporous CuO-TiO2 Nanotube Catalysts. *Catal.* **2017**, *7*, 129, doi:10.3390/catal7050129.
4. Wu, C.H.; Liu, C.; Su, D.; Xin, H.L.; Fang, H.-T.; Eren, B.; Zhang, S.; Murray, C.B.; Salmeron, M.B. Bimetallic synergy in cobalt–palladium nanocatalysts for CO oxidation. *Nat. Catal.* **2019**, *2*, 78–85, doi:10.1038/s41929-018-0190-6.
5. Nikolaev, S.; Golubina, E.; Shilina, M. The effect of H 2 treatment at 423–573 K on the structure and synergistic activity of Pd–Cu alloy catalysts for low-temperature CO oxidation. *Appl. Catal. B: Environ.* **2017**, *208*, 116–127, doi:10.1016/j.apcatb.2017.02.038.
6. Mozer, T.S.; Passos, F.B. Selective CO oxidation on Cu promoted Pt/Al2O3 and Pt/Nb2O5 catalysts. *Int. J. Hydrogen Energy* **2011**, *36*, 13369–13378, doi:10.1016/j.ijhydene.2011.08.011.
7. Sun, G.; Mu, X.; Zhang, Y.; Cui, Y.; Xia, G.; Chen, Z. Rare earth metal modified CuO/γ-Al2O3 catalysts in the CO oxidation. *Catal. Commun.* **2011**, *12*, 349–352, doi:10.1016/j.catcom.2010.10.013.
8. Devi, M.M.; Dolai, N.; Sreehala, S.; Mishra, R.S.K.; Sharma, S.; Biswas, K.; Jaques, Y.M.M.; Galvao, D.S.; Tiwary, C.S. Morphology controlled graphene–alloy nanoparticle hybrids with tunable carbon monoxide conversion to carbon dioxide. *Nanoscale* **2018**, *10*, 8840–8850, doi:10.1039/c7nr09688g.
9. AlKetbi, M.; Polychronopoulou, K.; Jaoude, M.A.; Vasiliades, M.A.; Sebastian, V.; Hinder, S.J.; Baker, M.A.; Zedan, A.F.; Efstathiou, A.M. Cu-Ce-La-Ox as efficient CO oxidation catalysts: Effect of Cu content. *Appl. Surf. Sci.* **2020**, *505*, 144474, doi:10.1016/j.apsusc.2019.144474.
10. Santos, V.P.; Carabineiro, S.A.; Tavares, P.B.; Pereira, M.F.; Órfão, J.J.; Figueiredo, J.L. Oxidation of CO, ethanol and toluene over TiO2 supported noble metal catalysts. *Appl. Catal. B: Environ.* **2010**, *99*, 198–205, doi:10.1016/j.apcatb.2010.06.020.

11. Satsuma, A.; Osaki, K.; Yanagihara, M.; Ohyama, J.; Shimizu, K.-I. Activity controlling factors for low-temperature oxidation of CO over supported Pd catalysts. *Appl. Catal. B: Environ.* **2013**, *132-133*, 511–518, doi:10.1016/j.apcatb.2012.12.025.
12. Cai, F.; Yang, L.; Shan, S.; Mott, D.; Chen, B.H.; Luo, J.; Zhong, C.-J. Preparation of PdCu Alloy Nanocatalysts for Nitrate Hydrogenation and Carbon Monoxide Oxidation. *Catal.* **2016**, *6*, 96, doi:10.3390/catal6070096.
13. Ahmad, Y.H.; Mohamed, A.T.; Hassan, W.M.; Soliman, A.; Mahmoud, K.A.; Aljaber, A.S.; Al-Qaradawi, S.Y. Bimetallic palladium-supported halloysite nanotubes for low temperature CO oxidation: Experimental and DFT insights. *Appl. Surf. Sci.* **2019**, *493*, 70–80, doi:10.1016/j.apsusc.2019.07.009.
14. Zhan, W.; Wang, J.; Wang, H.; Zhang, J.; Liu, X.; Zhang, P.; Chi, M.; Guo, Y.; Guo, Y.; Lu, G.; et al. Crystal Structural Effect of AuCu Alloy Nanoparticles on Catalytic CO Oxidation. *J. Am. Chem. Soc.* **2017**, *139*, 8846–8854, doi:10.1021/jacs.7b01784.
15. Fang, Y.; Chi, X.; Li, L.; Yang, J.; Liu, S.; Lu, X.; Xiao, W.; Wang, L.; Luo, Z.; Yang, W.; et al. Elucidating the Nature of the Cu(I) Active Site in CuO/TiO2 for Excellent Low-Temperature CO Oxidation. *ACS Appl. Mater. Interfaces* **2020**, *12*, 7091–7101, doi:10.1021/acsami.9b18264.
16. Harzandi, A.M.; Tiwari, J.N.; Lee, H.S.; Jeon, H.; Cho, W.J.; Lee, G.; Baik, J.; Kwak, J.H.; Kim, K.S. Efficient CO Oxidation by 50-Facet Cu2O Nanocrystals Coated with CuO Nanoparticles. *ACS Appl. Mater. Interfaces* **2017**, *9*, 2495–2499, doi:10.1021/acsami.6b13843.
17. Polychronopoulou, K.; AlKhoori, A.A.; Efstathiou, A.M.; Jaoude, M.A.; Damaskinos, C.M.; Baker, M.A.; Almutawa, A.; Anjum, D.H.; Vasiliades, M.A.; Belabbes, A.; et al. Design Aspects of Doped CeO2 for Low-Temperature Catalytic CO Oxidation: Transient Kinetics and DFT Approach. *ACS Appl. Mater. Interfaces* **2021**, *13*, 22391–22415, doi:10.1021/acsami.1c02934.
